# Understanding de novo onset of anxiety during COVID‐19: Pre‐pandemic socio‐emotional functioning in vulnerable children

**DOI:** 10.1002/jcv2.12076

**Published:** 2022-05-05

**Authors:** Dolapo Adegboye, Jessica Lennon, Olivia Batterbee, Anita Thapar, Stephan Collishaw, Katherine Shelton, Kate Langley, Christopher Hobson, Andrea Higgins, Stephanie van Goozen

**Affiliations:** ^1^ School of Psychology Cardiff University Cardiff UK; ^2^ School of Medicine Cardiff University Cardiff UK

**Keywords:** anxiety, emotion, peer relationships, resilience, self‐esteem

## Abstract

**Background:**

There is a need to understand and mitigate the psychological impacts of the COVID‐19 pandemic for children known to be vulnerable. Data from prior to the pandemic are required to provide robust assessments of the socio‐emotional impacts of COVID‐19 and identify those who are more vulnerable.

**Method:**

This study capitalises on an ongoing UK study of primary school children (4–8 years) identified prior to the pandemic as “at risk” for mental health problems by teachers. We collected mental health and social‐emotional functioning data prior to the pandemic (Time 1) and re‐assessed this cohort (*N* = 143) via researcher‐led videocalls during lockdown (Time 2, summer 2020) and post‐lockdown, 12 months later (Time 3; summer 2021).

**Results:**

Mental health problems, particularly clinically significant anxiety, increased from 34% to 43% during lockdown and to 48% post‐lockdown. Parental mental health difficulties (anxiety and depression) were prevalent during lockdown (40%) but had decreased 1 year later (20%). Children who developed clinically significant anxiety during the pandemic had impaired socio‐emotional functioning at Time 1 (i.e., impaired emotion recognition, low self‐esteem and social problems) and a high proportion (44%) had no contact with any peers during lockdown, which may have contributed to their anxiety, especially their school anxiety.

**Conclusion:**

The pandemic appears to have exacerbated anxiety in already vulnerable children. A profile of socio‐emotional problems identified a group of children who developed significant anxieties during the pandemic. These socio‐emotional processes can be targeted for intervention to mitigate the negative mental health consequences of the pandemic and contribute to resilience in children.

**Key points**

- Although there is evidence of the early impact of COVID‐19 and the associated social isolation on children and young people's mental health, there is a dearth of longitudinal research in which changes in mental health symptoms from a pre‐pandemic baseline to more than one timepoint during the pandemic, among those already identified as vulnerable pre‐COVID. The current study provides such evidence
- In vulnerable young children, clinically significant anxiety increased from pre‐pandemic to the summer of 2020 and remained high one year later despite a return to school and improvements in parental mental health
- Children who developed de novo or persistent anxiety during the pandemic had impaired emotion recognition and more peer relationship problems pre‐pandemic
- Easing restrictions and getting children back to school may not be sufficient to overcome the impacts of the lockdown and school closure in children who are vulnerable
- Targeted interventions to improve socio‐emotional processes in children identified as being at risk of mental health problems should be examined

There is growing evidence of the profound early impacts of the COVID‐19 lockdown and school closures on mental health functioning (Newlove‐Delgado et al., 2021). Bearing in mind that the risk of further waves of COVID‐19 and associated social control measures is high, there is a pressing need to understand the psychosocial consequences of the COVID‐19 pandemic and associated lockdown on primary school children, especially those known to be at risk of significant emotional, cognitive and behavioural problems, and to identify modifiable child, family and school‐related factors that contribute to risk and resilience (Holmes et al., 2020; Jefsen et al., 2020).

There is substantial heterogeneity in the way children cope with and respond to lockdown‐related experiences (Adegboye et al., 2021), with some children having fared better than expected (Cost et al., 2021). A better understanding of the immediate and ongoing psychological and social consequences for children and their families is needed in order to develop policies and interventions to mitigate the onset and exacerbation of mental health problems and provide tailored support for vulnerable groups of children.

Longitudinal studies that include assessments of mental health prior to the onset of the COVID‐19 pandemic are scarce, as are studies that include data collection at multiple timepoints during the pandemic (Breaux et al., 2021). In the UK, lockdowns and school closures applied to most children from March 2020 to the summer holiday of 2020; schools re‐opened in September 2020, closed again in January 2021 and re‐opened again in April 2021.

The current study focuses on a sample of children with pre‐existing mental health vulnerabilities and combines comprehensive pre‐COVID assessments of mental health and social context with multiple assessments during the COVID‐19 pandemic. This enables us to examine changes in and predictors of mental health functioning during the pandemic. We carried out intensive videocall assessments in a sample of 143 families and children in the summers of 2020 and 2021 to arrive at a detailed understanding of the specific mental health needs of these children and the economic profiles of their families, including whether and how these had changed because of the COVID‐19 pandemic.

In a first paper, reporting findings from an initial COVID wave of assessment in the summer of 2020 (Adegboye et al., 2021), we found that mental health problems, particularly anxiety, increased during the pandemic in children who were already vulnerable. We also found that parent mental health problems and financial stress (e.g., lost employment, loss of income, inability to pay bills) during lockdown were highly prevalent, especially amongst low‐income families, and that financial strain indirectly predicted increases in child mental health problems through parental mental health problems. Because that paper reported results from a single wave of COVID assessment, it is unclear whether the observed changes reflected short‐lived responses to the immediate consequences of the first lockdown or were signs of more enduring changes in mental health.

In the current paper, we report findings from three assessment waves (pre‐pandemic, summer of 2020, and summer of 2021) with the aim of identifying modifiable child‐related risk and protective factors that contribute to variability in mental health outcomes. In particular, we examined whether pre‐pandemic psychological processes and social difficulties, known to index vulnerability for mental health problems, were associated with changes in child anxiety. We focused on child emotion recognition and self‐esteem, and teacher‐reported social problems. The lockdown resulted in school closures and children's isolation from their peers. Children therefore had fewer and limited opportunities to achieve and to interact with others. Although all children were affected by this experience, it may have triggered de novo onset of anxiety in some vulnerable children.

## CHILD SELF‐ESTEEM AND EMOTION RECOGNITION AS RISK FACTORS FOR ANXIETY

Emotion recognition and self‐esteem are psychological processes that are known to be associated with anxiety and poor mental health and are also likely to be affected by social isolation. Correctly recognising and understanding others' emotions is fundamental to forming and maintaining effective social relationships and to psychological adjustment (Carpendale & Lewis, 2015). Accurate recognition of emotional expressions from facial cues is associated with positive social relationships, social competence, and peer acceptance (Denham et al., 2014). Conversely, impairments in emotion recognition are associated with social rejection, victimisation, adjustment problems and anxiety symptoms in childhood (Richards et al., 2007). Emotion recognition is a transdiagnostic process: different types of psychopathology have been linked to a tendency to incorrectly attribute emotions (Van Goozen et al., 2022). Social isolation during school closures may have deprived children of the opportunity to develop and strengthen this important skill; returning to school and re‐joining peer groups after closures ended may also have posed challenges for those with impaired emotion recognition.

Self‐esteem is another important indicator of psychological functioning (McCauley et al., 2017). People with high self‐esteem are generally happier and more likely to enjoy close friendships; low self‐esteem has been implicated in a variety of mental health problems and is a strong predictor of emotional and behavioural problems (Keane & Loades, 2017). Self‐esteem is lower in those with impairments in emotion recognition (Wells et al., 2020).

In the current study we examine whether a pre‐pandemic profile of low socio‐emotional functioning constituted a risk factor for the deterioration of mental health and onset of anxiety problems during the pandemic. Our first aim was to examine changes in mental health in vulnerable children during COVID‐19. Our second aim was to examine whether pre‐COVID socioemotional functioning could predict (a) increases in child anxiety, and (b) risk of new onset anxiety during the pandemic. Specifically, we investigated whether children who exhibited low self‐esteem, impaired emotion recognition and peer problems, but no anxiety, pre‐COVID, developed clinically significant anxiety during the pandemic because of their greater vulnerability to the effects of the lockdowns and social isolation, and being less well prepared for the eventual return to school.

## METHOD

### Sample

The participants were 143 children (aged 4–8 years at initial assessment; mean age = 6 years 2 months, SD = 1.06, and aged 5–10 years at the time of our first assessment during lockdown; mean age = 7 years 8 months, SD = 1.14; 38% girls) previously referred by teachers or Special Educational Needs Co‐ordinators (SENCOs) because of emotional, cognitive, or behavioural problems exhibited in the classroom. The children came from 67 mainstream primary schools in the UK. Following their referral, these 143 children and families had been assessed (i.e., pre‐COVID, Time 1) using detailed and well‐established face‐to‐face interview and questionnaire assessments of child mental health, social and family risk and protective factors, and RDoC informed laboratory task‐based assessments of socio‐emotional and cognitive processes and functioning.^1^ Children and families were contacted again during COVID and assessed via videocall during the first lockdown (summer 2020; Time 2) and 12 months later at the end of the school year (summer 2021; Time 3). All procedures were approved by the relevant institutional ethics committee (EC.20.06.09.6053RA). Written informed consent was obtained from a parent or caregiver for each child who participated in the assessment.

### Measures

#### Child mental health

The SDQ is a 25‐item screening questionnaire for mental health difficulties in children and young people aged 3–16 years (Goodman, 1997). Teachers and parents assessed children using the Strengths and Difficulties Questionnaire [SDQ] (Goodman, 1997) when the child was first assessed (pre‐COVID, Time 1); parents rated the children again using the SDQ at Time 2 and 3. The questionnaire consists of five subscales (emotional symptoms, hyperactivity/inattention, conduct problems, peer problems, and prosocial behaviour). A total difficulties score (SDQ total), comprising the first four subscale scores, indicates the overall extent of a child's mental health problems. We categorised the subscales according to their recommended cut‐off points (Goodman et al., 2010) indicating a high/very high score. Where there were missing scores, scale means were calculated from the items with valid scores. Table 2 shows the parent SDQ ratings across the three times. Teacher SDQ ratings of the children at time of referral (Time 1) are also reported (see Table 1). Teacher ratings of child peer problems were used in our analysis of child socio‐emotional functioning; higher scores on the SDQ peer problems subscale are reflective of interpersonal problems.

**TABLE 1:** Percentage of children scoring above SCARED cut‐off at each of the three times of assessment (*N* = 143)

	Time 1	Time 2	Time 3
	Pre‐Covid	Summer 2020	Summer 2021
Above SCARED cut‐off (*≥* 25)	34%	43%	48%

The Screen for Child Anxiety Related Emotional Disorders (SCARED; Birmaher et al., 1999) is a reliable and valid way of screening for diagnosed anxiety in clinical and community samples (Runyon et al., 2018). The SCARED consists of 41 questions; responses are recorded by the parent as describing the child's behaviour during the previous 3 months. Where there were missing scores, scale means were calculated from the items with valid scores. A total score of ≥25 suggests the presence of an anxiety disorder. Subscales assess five specific anxiety disorders: Panic/Somatic Anxiety (PD), General Anxiety (GAD), Separation Anxiety (SAD), Social Phobia (SP) and School Phobia (ScP). Items for each subscale of the SCARED measure revealed good internal consistency (ranging from 0.60 to 0.81). Parents completed the SCARED three times, pre‐COVID (Time 1) and again in the summers of 2020 (Time 2) and 2021 (Time 3).

Different anxiety groups were created for additional analyses using total SCARED scores at the three timepoints. Only children whose anxiety symptoms were reported pre‐Covid, and at the following two timepoints were included. The number of children with below cut‐off and above cut‐off anxiety levels is shown in Table 1. Children were grouped according to whether they had exhibited persistently low levels of anxiety, persistently high levels of anxiety, new onset anxiety, reduced anxiety, or were inconsistent. These classifications were used to explore all potential trajectories that could occur over the 3 time points. The ‘consistently low’ anxiety group (*n* = 63) included children who had scores below the cut‐off (total score <25) at all 3 time‐points (*M*_time1_ = 10.094; *M*_time2_ = 12.873; *M*_time3_ = 11.524). The ‘consistently high’ anxiety group (*n* = 32) comprised children who had scores above the cut‐off levels (total score ≥25) at all the 3 time‐points (*M*_time1_ = 39.169; *M*_time2_ = 44.733; *M*_time3_ = 45.497). The ‘new onset’ anxiety group (*n* = 27) consisted of children who had scores below the cut‐off at time 1 but above the cut‐off at times 2 and 3 (*M*_time1_ = 13.125; *M*_time2_ = 30.323; *M*_time3_ = 33.202). The reduced anxiety group (*n* = 7) included children who had scores above the cut‐off at time 1 but below the cut‐off at Times 2 and 3. Lastly, the inconsistent group (*n* = 14) comprised of children whose scores were more variable (e.g., below cut‐off at Time 1, above cut‐off at Time 2, below cut‐off at Time 3). Due to the small number of children in the reduced anxiety and inconsistent groups, these were excluded from further analyses involving anxiety.

### Parental mental health: Anxiety and depression

The Hospital Anxiety and Depression Scale (HADS; Zigmond & Snaith, 1983) is a 14‐item screening measure designed to assess symptoms of anxiety and depression in non‐psychiatric populations, identifying individuals at elevated risk for anxiety and depressive disorders. Scores range from 0 to 21, with scores from 8 to 10 indicating borderline levels and scores from 11 to 21 indicating abnormal levels warranting clinical assessment. Parents completed the HADS in the summer of 2020 (Time 2) and 2021 (Time 3). There was good internal consistency for both subscales (with Cronbach's alphas ranging from 0.73 to 0.83 at Time 2 and 0.65 to 0.82 at Time 3).

### Socio‐emotional functioning

#### Self‐esteem

The Self Perception Profile for Children (SPPC; Harter, 1982) is a self‐report questionnaire that assesses how children evaluate themselves in different areas of their life, including self‐perceived social and cognitive competence. The SPPC is a valid, reliable measure and the most widely used questionnaire for assessing self‐esteem in children (Muris et al., 2003). The current study used a total self‐esteem score (SPPC total) comprising three subscale scores (self‐perception of cognitive competence, physical competence, and social acceptance amongst peers) to indicate overall self‐esteem. Each subscale contains six items, and all items are scored on a scale of one to four where one reflects the lowest perceived adequacy and four reflects the highest. Cronbach's alpha was 0.70. The SPPC was completed by the children at Time 1.

### Facial emotion recognition

Emotion recognition was examined using the facial emotion recognition test (FER; Hunnikin et al., 2021; Wells et al., 2020) consisting of 40 faces chosen from the Radboud Faces Database (Langner et al., 2010) depicting happy, sad, fearful, angry, and neutral facial expressions. Varying intensity versions of each affective expression were created by merging the target expression (100%) with a neutral expression (0%) to create expressions that varied between 90% and 35%. Each facial expression was presented for 3s before the emotion category labels appeared and the child was asked to identify the facial expression. Raw FER data were transformed to calculate a negative response bias variable, representing a child's tendency to incorrectly recognise negative emotional expressions or to categorise neutral faces as negative (Chronaki et al., 2013). The FER has good reliability (*α* = 0.83; Airdrie et al., 2018) and was completed by children at Time 1.

### Social contact during lockdown

Parents/carers were asked about the frequency with which their children had contact with friends outside of the home during lockdown and school closure. Responses were given on a 7‐point scale ranging from ‘not at all’ to ‘most of the day’ for different means of contact including, phoning, video‐talking, and face‐to‐face. Responses were converted into a dichotomous score of either ‘no contact by any type’ (responses of ‘not at all’ for all modes of communication) or some contact of at least one type (any response between ‘once per week’ to ‘most of the day’).

### Intellectual ability

Cognitive ability was assessed with the computerized Lucid Ability assessment (Singleton, 2001). The validity of this test is comparable to a range of conventional IQ measures, including the Wechsler Intelligence Scale for Children (WISC‐III), the British Ability Scales (Second Edition) and the British Picture Vocabulary Scale (Singleton, 2001). The test was completed by children at Time 1.

### Procedure

At baseline (pre‐COVID, Time 1), parents visited our research unit and provided child and family background information (see Table 2). The sample assessed and reported on during COVID (Time 2 and Time 3) was a subset of a larger sample of children who had been referred by schools prior to the onset of COVID. While all eligible families were contacted, time constraints between the receipt of funding (mid‐July 2020) and children's return to school (mid‐September 2020) meant that we were able to interview 143 children and families to assess the effects of the lockdown, school closure and social isolation (summer of 2020, Time 2). This sample was re‐interviewed 1 year later (summer of 2021; Time 3). No significant differences were found on any sociodemographic, parent or child mental health measures, including teacher referral (teacher SDQ; Table 2) between those who participated (*n* = 143) in the study to assess the effects of COVID and those who did not participate.

**TABLE 2:** Participant demographics at baseline (pre‐COVID) (*n* = 143)

	Percentage
***Socioeconomic indicators***	
WIMD Quintiles^a^ (two most deprived categories)	47
Income (less than £20,000 pa)	29
Families including a keyworker	55
***Parental education (highest)***	
No formal educational qualification	8
O‐levels/GCSEs	29
A‐levels/Higher	23
University degree	19
Higher or postgraduate degree	21
***Ethnicity***	
British	76
British/European	2
Other European	1
British/Bangladeshi/Indian/Pakistani	1
British/African	1
British/Caribbean	1
British/Turkish	1
British/Arabian	1
Arab	1
Other	4
Unknown	9
***Child adversity***	
Physical abuse present	49
Parental separation	18
Parental mental health problems	42
Parental incarceration	5
Child adversity sum (≥1)	69
***Support***	
Social services involvement	29
CAMHS involvement	17
Extra school support for SEN	63
***Teacher‐reported SDQ***	
SDQ total, mean (SD)	15.85 (6.74)
Percent high/very high	51
SDQ emotional, mean (SD)	2.52 (2.34)
Percent high/very high	23
SDQ conduct, mean (SD)	3.06 (2.58)
Percent high/very high	39
SDQ hyperactivity, mean (SD)	7.17 (2.96)
Percent high/very high	57
SDQ peer, mean (SD)	3.04 (2.34)
Percent high/very high	31
SDQ prosocial, mean (SD)	4.92 (2.94)
Percent high/very high	36
***Lucid Verbal and Nonverbal IQ***	
Verbal standard score, mean (SD)	105.62 (16.03)
Nonverbal standard score, mean (SD)	94.17 (17.06)

^a^ Welsh Assembly Government (2019). *Welsh IMD 2019 data.* Available from: https://statswales.gov.wales/Catalogue/Community‐Safety‐and‐Social‐Inclusion/Welsh‐Index‐of‐Multiple‐Deprivation.

At Time 1 exposure to severe childhood adversity was indexed by parent's reports of whether the child had ever experienced physical abuse, parental separation, parental mental health problems, and parental incarceration. Parents also reported on any current involvement of social or child and adolescent mental health services, and any extra support provided by the child's school for special educational needs. Measures of child mental health (SDQ, SCARED) were also completed by parents (see Table 2). Children completed the SPPC and FER.

To assess the effects of COVID on child mental health parents and children participated in researcher‐led videocalls (mean interview duration 1.5 h) at Time 2 (summer of 2020) and Time 3 (summer of 2021). During these calls parents again completed measures of child mental health (SDQ, SCARED), but also completed measures of parental anxiety and depression (HADS). COVID‐19 exposure (risk) and current infection status were assessed, as well as school provision and contact; change in employment status and financial stress; daily routine and lifestyle; and time spent educating children and engaging with social contacts.

## DATA ANALYSIS

Data analyses were conducted using SPSS 26.0 (IBM Corp, 2019). Prior to the main statistical analyses, the distributions of the key measures (i.e., SDQ, SCARED, HADS, SPPC, FER) were examined and found to be normal. Next, descriptive statistics for the sample were calculated. Repeated measures ANOVAs and follow‐up post‐hoc tests were used to assess change in mental health functioning over time. Bonferroni corrections were applied to all multiple statistical tests to calculate adjusted *p* values.

Correlational analysis was used to examine the relationships between measures of child and parent mental health and multiple linear regression analysis was used to examine the capacity of children's socioemotional functioning at Time 1 (i.e., teacher‐rated peer problems, social contact during the lockdown, negative emotion response bias, and self‐esteem) to predict change in child anxiety from Time 1 (pre‐COVID) to Time 3 (post‐lockdown). Children's Time 3 SCARED total scores were regressed on the measures of children's socioemotional functioning, controlling for children's Time 1 SCARED total scores. Finally, multinomial logistic regression analysis was conducted to examine whether children's socioemotional functioning at Time 1 predicted whether children exhibited persistently low, persistently high, or new onset anxiety symptoms. The Box‐Tidwell (1962) procedure was used to assess the linearity of the predictors with respect to the logit of the outcome variable (trajectory group). All continuous outcome variables were found to be linearly related to the logit of the dependent variable. Continuous predictors were also standardised to facilitate interpretation of the regression weights.

## RESULTS

Demographic data for the sample are shown in Table 2.

Teachers' ratings on the SDQ showed that 51% of the children were at high or very high risk of mental health problems when they were initially referred (see Table 2). More specifically, around one‐third of the children were at high or very high risk of conduct and/or peer problems, and low in prosocial behaviour; over half of the children were at high or very high risk of problems with attention and hyperactivity. The socioeconomic characteristics of our sample also indicate that many of the children's families can be regarded as vulnerable. Levels of deprivation and poverty are relatively high, two‐thirds of the children had special educational needs, nearly one‐third of the families were receiving support from social services, and 69% of the children had experienced at least one of four forms of serious adversity.

### Changes in child and parent mental health

Table 3 shows the descriptive statistics and results of repeated measure ANOVAs for the measures of child and parental mental health at each of the three timepoints, where scores for these measures are available (although the HADS was administered at Time 1, there was a low completion rate [*n* = 83] at this timepoint, due to time restrictions).

**TABLE 3:** Descriptive statistics and repeated measure ANOVAs for child and parent mental health symptoms

	Time 1	Time 2	Time 3			
	Mean (SD)	Mean (SD)	Mean (SD)	Statistics		
**SDQ**				*F*	*P*	ηp^2^
*Total*	18.32 (6.92)	19.30 (6.77)	19.00 (7.42)	2.61	0.075	0.018
*Emotional*	3.70 (2.70)^b^	4.00 (2.61)^c^	4.48 (2.75)^b^ ^,^ ^c^	7.09**	0.001	0.048
*Conduct*	4.16 (2.75)^b^	4.04 (2.66)	3.70 (2.73)^b^	4.33*	0.014	0.030
*Hyperactivity*	7.44 (2.65)	7.80 (2.35)	7.53 (2.47)	2.85	0.060	0.020
**SCARED**						
*Total*	20.22 (14.93)^a^ ^,^ ^b^	24.18 (15.26)^a^	25.02 (16.16)^b^	13.80**	<0.001	0.089
*Panic/somatic*	3.37 (4.53)^a^ ^,^ ^b^	7.69 (5.58)^a^	7.57 (5.47)^b^	76.10**	<0.001	0.349
*GAD*	5.45 (4.47)^b^	6.09 (3.73)	6.50 (4.07)^b^	6.02**	0.003	0.041
*Separation*	5.04 (3.96)^a^	4.20 (3.24)^a^	4.55 (3.59)	4.68**	0.010	0.032
*School*	1.28 (1.58)^a^ ^,^ ^b^	2.48 (1.94)^a^	2.65 (2.01)^b^	43.07**	<0.001	0.233
*Social*	5.08 (3.97)^a^ ^,^ ^b^	3.71 (2.89)^a^	3.74 (3.02)^b^	14.82**	<0.001	0.094
**HADS**				*t*	*p*	*d*
*Total*	‐	14.20 (7.75)	11.35 (7.34)	4.55**	<0.001	0.412
*Anxiety*	‐	7.73 (4.73)	6.35 (3.83)	3.73**	<0.001	0.336
*Depression*	‐	6.49 (4.06)	5.02 (4.33)	3.90**	<0.001	0.353

Abbreviations: HADS, Hospital Anxiety and Depression Scale; SCARED, Screen for Child Anxiety Related Emotional Disorders; SDQ, Strengths and Difficulties Questionnaire.

^a^ Significant difference between Time 1 and Time 2.

^b^ Significant difference between Time 1 and Time 3.

^c^ Significant difference between Time 2 and Time 3.

* = *p* < 0.05; ** = *p* < 0.01.

Regarding child mental health, there was a significant increase in SDQ emotional problems between Times 1 and 2, but no change between Times 2 and 3. By contrast, SDQ conduct problems decreased significantly between Time 1 and Time 3. There were also significant changes in child anxiety (SCARED), including significant increases in the panic/somatic and school anxiety between Times 1 and 2, increases that were sustained at Time 3, but significant reductions in separation anxiety and social anxiety between Times 1 and 2, reductions that were also sustained at Time 3.

Regarding parent mental health, there were relatively high levels of problems, particularly anxiety, at Time 2, but significantly lower levels at Time 3 (*ps* < 0.001). Prevalence of parental mental health difficulties (anxiety and depression) during lockdown (Time 2) was 40% but this had decreased 1 year later (Time 3) to 20%.

### Associations between child and parent mental health

The relevant correlation coefficients are shown in Table 4. It is evident that SDQ and SCARED scores were relatively strongly correlated across the three measurement waves. Also apparent are the significant correlations between parental mental health and the measures of child mental health.

**TABLE 4:** Correlations between measures of child and parent mental health across three times of assessment (*n* = 143)

Variable	*n*	*Mean*	*SD*	1	2	3	4	5	6	7	8
1. SDQ total (Time 1)	142	18.32	6.92	‐							
2. SDQ total (Time 2)	142	19.26	6.76	0.709**	‐						
3. SDQ total (Time 3)	142	19.01	7.40	0.705**	0.764**	‐					
4. Child anxiety (Time 1)	142	20.22	14.93	0.336**	0.172*	0.177*	‐				
5. Child anxiety (Time 2)	142	24.18	15.26	0.389**	0.480**	0.330**	0.602**	‐			
6. Child anxiety (Time 3)	142	25.02	16.16	0.366**	0.416**	0.460**	0.645**	0.791**	‐		
7. Parental anxiety/depression (Time 2)	128	14.12	7.71	0.263**	0.454**	0.310**	0.237**	0.361**	0.251**	‐	
8. Parental anxiety/depression (Time 3)	133	11.05	7.23	0.240**	0.379**	0.274**	0.239**	0.340**	0.288**	0.543**	‐

*Note*: HADS, Hospital Anxiety and Depression Scale; SCARED, Screen for Child Anxiety Related Emotional Disorders; SDQ, Strengths and Difficulties Questionnaire.

*p < 0.05; **p < 0.01.

### Does impaired socio‐emotional functioning at Time 1 predict onset of clinical anxiety during the pandemic?

The three anxiety groups (‘persistently low’, ‘persistently high’ and ‘new onset’) were compared with respect to children's pre‐COVID socio‐emotional functioning. Demographic characteristics of the three groups can be found in Table 5. There were no significant differences between the three groups on any of these demographic variables, but the ANOVAs reported below nevertheless controlled for age and IQ.

**TABLE 5:** Descriptive statistics and comparisons between anxiety groups

	Consistently low (*n* = 63)	Consistently high (*n* = 32)	New onset (*n* = 27)
**Demographic measures**			
Mean age in months (*SD*)	75.41 (12.12)	76.16 (12.90)	71.67 (11.65)
Percent girls	36	48	33
Mean verbal IQ (*SD*)	106.25 (17.73)	106.83 (13.51)	101.38 (13.56)
Mean nonverbal IQ (*SD*)	94.86 (19.14)	95.42 (15.21)	96.38 (12.82)
**Socioemotional measures**			
Teacher‐rated peer problems (*SD*)	3.00^a^ (2.38)	2.12^b^ (1.82)	3.95^a^ (2.50)
Self‐esteem (*SD*)	57.98 (6.59)	57.95 (6.48)	53.70 (7.26)
Negative response bias (*SD*)	0.14 (0.08)	0.14 (0.07)	0.20 (0.13)
**Social contact during lockdown/school closure**			
Percent having no contact through any mode of communication	27	20	44

*Note*: Means not sharing a common superscript differ significantly (*p* < 0.05, Bonferroni‐corrected).

Also shown in Table 5 are the children's mean scores on the socioemotional measures at Time 1, for each of the three anxiety groups. Controlling for the effects of age and IQ, there are significant differences between the three groups with respect to teacher‐rated peer problems *F*(2, 98) = 3.35, *p* = 0.039, ηp^2^ = 0.064, self‐esteem, *F*(2, 96) = 3.10, *p* = 0.049, ηp^2^ = 0.061, and negative emotion response bias, *F*(2, 97) = 3.12, *p* = 0.049, ηp^2^ = 0.060. The new onset anxiety group differed significantly from the consistently high group with respect to teacher‐rated peer problems. Other pairwise comparisons were not significant.

Multiple linear regression was used to examine the capacity of the socioemotional measures to predict child anxiety at Time 3 while controlling for anxiety at Time 1, as well as age and IQ. The results are summarised in Table 6, where negative response bias to facial expressions was the only significant predictor of Time 3 anxiety, alongside Time 1 anxiety.

**TABLE 6:** Predictors of child anxiety at Time 3, controlling for age, IQ, and anxiety at Time 1

Predictors	B	*Beta*	*t*	*P*
*Model 1*				
Age	−0.003	−0.073	−0.721	0.472
Verbal IQ	−0.002	−0.069	−0.843	0.401
Nonverbal IQ	−0.001	−0.021	−0.211	0.833
Anxiety at Time 1	0.550	0.520	6.356	<0.001
*Model 2*				
Age	−0.002	−0.045	−0.445	0.657
Verbal IQ	−0.001	−0.051	−0.626	0.533
Nonverbal IQ	0.000	−0.007	−0.074	0.941
Anxiety at Time 1	0.579	0.548	6.699	<0.001
Teacher‐rated peer problems	0.010	0.046	0.552	0.582
Negative response bias	1.030	0.188	2.264	0.026
Self‐esteem	−0.002	−0.002	−0.256	0.798

*Note*: Model 1: *R* ^2^ = 0.269, *F*(4, 112) = 10.308, *p* < 0.001. Model 2: *R* ^2^ = 0.309, *F*(7, 109) = 6.958, *p* < 0.001.

Abbreviations: FER, Facial Emotion Recognition test; SCARED, Screen for Child Anxiety Related Emotional Disorders; SDQ, Strengths and Difficulties Questionnaire; SPPC, Self Perception Profile for Children.

Next, to ascertain the socioemotional predictors of new onset clinical level anxiety, we conducted multinomial logistic regression analysis in which ‘new onset anxiety’ served as the reference group. The Time 1 socioemotional variables (teacher‐rated peer problems, negative response bias to facial expressions, and self‐esteem) were entered as predictors, together with the social contact measure. The fit of this logistic regression model was significant, χ^2^(8) = 17.90, *p* = 0.022, showing that the predictors collectively significantly predicted anxiety group membership. The model explained 17.8% (Nagelkerke *R* ^*2*^) of the variance in anxiety status and correctly classified 59.8% of the cases.

The results for the individual predictors are summarised in Table 7. Different socioemotional variables significantly distinguished between the consistently low anxiety group and the new onset group, on the one hand, and the consistently high anxiety group and the new onset anxiety group, on the other. Whereas negative response bias to facial expressions significantly distinguished the consistently low anxiety group and the new onset group, teacher‐rated peer problems significantly distinguished the consistently high anxiety group and the new onset group.

**TABLE 7:** Predictors of anxiety group (consistently high, consistently low, new onset), with new onset group as reference category

Predictors	Parameter estimates			
	*B*	*SE B*	*Wald*	*p*
*Consistently low*				
Teacher‐rated peer problems	−0.165	0.124	1.774	0.183
Negative response bias	−6.062	2.896	4.382	0.036
Self‐esteem	0.025	0.040	0.382	0.537
Social contact during lockdown	1.018	0.566	3.242	0.072
*Consistently high*				
Teacher‐rated peer problems	−0.346	0.147	5.567	0.018
Negative response bias	−6.098	3.360	3.295	0.069
Self‐esteem	−0.013	0.046	0.075	0.785
Social contact during lockdown	1.163	0.675	2.972	0.085

## DISCUSSION

Previous research on the mental health consequences of the COVID pandemic has tended to focus on older children and adolescents, reporting increases in anxiety and emotional disorders (Barendse et al., 2021; Newlove‐Delgado et al., 2021). The current study is one of the first to combine comprehensive pre‐COVID assessment in young children who were already at risk with data collection at multiple timepoints during the pandemic, enabling us to examine changes in mental health in these children.

Although causal conclusions cannot be drawn in the absence of a ‘no‐COVID’ control group, the results suggest that the lockdowns, the associated school closures, and the loss of social contact resulting from the pandemic had a significant and negative impact on these children's mental health, especially their anxiety. Clinically significant anxiety increased from pre‐COVID to the lockdown of summer 2020 and remained high in the summer of 2021, after children returned to school. Although there was evidence that parental anxiety and depression were also high during lockdown, these were lower after lockdown, 1 year later. Returning to employment and the availability of greater social support, two factors that were mentioned by parents during the interviews, could explain these findings.

Our assessment of child anxiety revealed significant increases in generalized anxiety, panic and somatic symptoms, and school anxiety, but reductions in separation and social anxiety. It seems likely that children's exposure to parental and media discussions about the risks of illness and death, coupled with the need to take precautionary measures, made children more aware of their bodily sensations and more concerned about getting ill and/or dying, perhaps giving rise to panic. Worries about home‐schooling and schoolwork, not being at school or returning to school are likely reasons for the increase in school anxiety. The data show that these anxieties did not decrease when the period of social isolation ended, consistent with the view that once anxiety symptoms are present, regardless of the initial trigger, they can become self‐perpetuating (Ellis & Hudson, 2010).

We also aimed to identify modifiable child‐related factors that contributed to the risk of developing anxiety during the pandemic. We focused on children's pre‐pandemic socio‐emotional functioning and examined whether children's emotion recognition and self‐esteem, processes known to be associated with mental health, were linked to changes in anxiety levels over time. School closures and the resulting social isolation may have negatively impacted children's self‐esteem, especially their confidence in their academic abilities and their sense of being accepted by others.

There was evidence that a pre‐COVID profile of problematic peer relations and negatively biased emotion recognition constituted a risk factor for the onset of anxiety problems during COVID. Each of these factors emerged as differing significantly between the anxiety groups (consistently low, consistently high, new onset), as predicting anxiety levels over time, or as predicting the anxiety group to which children belonged. We know from what the children told us that being with friends is what helps them to be happy at school, and that social difficulties are significant factors in making them feel sad, scared, or angry at school.

Children with pre‐existing difficulties in socio‐emotional functioning may have been not only more vulnerable to the effects of the lockdowns and social isolation, but also less able to cope with the demands of returning to school, and as a result experienced greater anxiety. The social isolation experienced during lockdown would have deprived these children of opportunities to develop their social‐emotional skills and being away from school is likely to have reduced their confidence in their academic abilities and their sense of being socially accepted. Returning to school and re‐joining peer groups following school closures may have posed challenges for children with problematic peer relations and impaired emotion recognition, especially if they had no contact with their peers (which was the case for 44% of the children who developed new anxiety). Children told us that the necessary ‘bubbling’ of children and the resulting separation from their friends were additional setbacks they encountered. Furthermore, the additional challenges to vulnerable children and their families posed by having to engage with distance learning, coupled with the likelihood that social difficulties and social‐class‐based academic inequalities have been exacerbated upon return to school (Goudeau et al., 2021), may have increased anxiety and may have had a particularly negative impact on the wellbeing and academic performance of children who were already vulnerable (Arslan, 2018).

It should be acknowledged that our study also had certain limitations. First, although our measure of anxiety, the SCARED, is an established way of screening for childhood anxiety in clinical and community samples (Runyon et al., 2018), and is well suited to use in research contexts, especially one involving videocall data collection, it is not a formal measure of anxiety disorder.

Secondly, although our sample provided a rare opportunity to study trends in anxiety symptoms from before to during the COVID‐19 pandemic in vulnerable children, it is relatively small and does not allow the use of trajectory modelling techniques such as growth mixture modelling to identify subgroups and changes in anxiety across time. The sample was also specific in terms of age range, selection process (i.e., teacher referral) and being referred for a range of problems rather than specifically for clinical anxiety, and the findings may therefore not be readily generalisable to other clinical populations or the general population.

A third point is that although we collected data before and during the pandemic and were therefore able to examine change in mental health in this high‐risk sample over time, there was (of necessity) no comparison group of children who had not been exposed to the pandemic and the socio‐emotional risk factors were only measured once. As a result, we cannot draw firm conclusions about the effects of the pandemic and the role of impaired socio‐emotional functioning on children's anxiety.

## IMPLICATIONS AND FUTURE RESEARCH

Identifying modifiable child‐related factors helps us to understand why some children were more at risk of developing new or more intense anxiety during the pandemic and suggests ways of intervening that would improve children's mental health. If children who are at risk of developing severe anxiety were identified early, they could be helped to develop more positive social and emotional skills by focusing on psychological processes that are amenable to change. Selected and indicated prevention of emotional problems is more effective than universal prevention. For example, children identified by schools as having specific problems could be given training to improve their emotion recognition (Hunnikin et al., 2021). This is feasible and leads to improvement in mental health and wellbeing (Wells et al., 2021). However, there is a need for further trials to evaluate how effective such interventions are in improving the mental health of high‐risk children (Van Goozen, Langley & Hobson, 2022).

In conclusion, there is a pressing need to understand the psychological and social impacts of the COVID‐19 pandemic and associated lockdown on primary school children, especially those already known to be at risk of significant emotional and behavioural problems (Jefsen et al., 2020). We found that there was no improvement in child mental health (SDQ and anxiety) after returning to school, despite significant improvements in parental mental health in the same period. This suggests that returning to school may not be sufficient to address the impacts of the pandemic on children's mental health. The increase in children's mental health problems is unlikely to be a transitory phenomenon and addressing this issue will require ongoing support. The mental health gap between advantaged and disadvantaged children has not reduced during the last 20 years (Collishaw et al., 2019) and is likely to have increased during the pandemic. The major challenges that vulnerable children and their families already face, coupled with the likelihood that social difficulties and social‐class‐based academic inequalities have been exacerbated by the pandemic (Goudeau et al., 2021), is likely to have a negative impact on children's future wellbeing and academic performance. Our results indicate that children's pre‐pandemic socio‐emotional functioning is associated with the increased anxiety problems experienced by children during and after the period of lockdown. These psychological processes could be targeted for intervention in vulnerable children and be the focus of efforts to mitigate mental health problems and to strengthen resilience among children in general.

## CONFLICTS OF INTEREST

Kate Langley is a member of the Editorial Advisory Board for JCPP *Advances*. The remaining authors have declared that they have no competing or potential conflicts of interest. [Corrections made on 1 June 2022 after first online publication: The Conflicts of Interest statement has been updated.]

## ETHICS STATEMENT

All procedures were approved by the relevant institutional ethics committee (EC.20.06.09.6053RA). Written informed consent was obtained from a parent or caregiver for each child who participated in the assessment.

## AUTHOR CONTRIBUTIONS

**Dolapo Adegboye:** Conceptualization; Data curation; Formal analysis;Investigation; Methodology; Project administration; Supervision; Validation; Visualization; Writing – original draft. **Jessica Lennon:** Formal analysis; Investigation; Validation; Visualization. **Olivia Batterbee:** Formal analysis; Investigation; Validation; Visualization. **Anita Thapar:** Conceptualization; Funding acquisition; Supervision; Validation; Writing – review & editing. **Stephan Collishaw:** Conceptualization; Funding acquisition; Supervision; Validation; Writing – review & editing. **Katherine Shelton:** Conceptualization; Funding acquisition; Supervision; Validation; Writing – review & editing. **Kate Langley:** Conceptualization; Funding acquisition; Supervision; Validation; Writing – review & editing. **Christopher Hobson:** Conceptualization; Funding acquisition; Methodology; Supervision; Validation; Writing – review & editing. **Andrea Higgins:** Conceptualization; Investigation; Methodology; Supervision; Writing – review & editing. **Stephanie van Goozen:** Conceptualization; Data curation; Formal analysis; Funding acquisition; Investigation; Methodology; Project administration; Resources; Supervision; Validation; Visualization; Writing – original draft; Writing – review & editing.

## Data Availability

The data that support the findings of this study are available from the corresponding author upon reasonable request.

## ENDNOTE

^1^

http://www.cardiff.ac.uk/research/explore/research‐units/neurodevelopment‐assessment‐unit
